# Innate immune pathway activated mesenchymal stromal cells improve function and histologic outcomes in a rodent osteoarthritis model

**DOI:** 10.3389/fbioe.2025.1525969

**Published:** 2025-05-22

**Authors:** Cody Plaisance, Lyndah Chow, Renata Impastato, Zoë J. Williams, Isabella Sabino, Katie J. Sikes, Kelly S. Santangelo, Steven Dow, Lynn M. Pezzanite

**Affiliations:** ^1^ Department of Clinical Sciences, College of Veterinary Medicine and Biomedical Sciences, Colorado State University, Fort Collins, CO, United States; ^2^ Department of Microbiology, Immunology and Pathology, College of Veterinary Medicine and Biomedical Sciences, Colorado State University, Fort Collins, CO, United States

**Keywords:** osteoarthritis, mesenchymal stromal cell, regenerative therapies, STING, interferon

## Abstract

**Introduction:**

Intra‐articular administration of mesenchymal stromal cells (MSC) has demonstrated anti‐inflammatory and chondroprotective activity in both preclinical models and in randomized clinical trials in patients with osteoarthritis (OA). Nonetheless, precedent from MSC studies in non‐OA models suggests that the overall anti-inflammatory effectiveness of MSC can be improved by prior immune activation through cytokines or innate immune pathways.

**Methods:**

Therefore, in the current study, we determined whether activation of MSC by two different innate immune pathways (Toll‐like receptor 3 (TLR3) pathway or Stimulator of Interferon Genes (STING) pathway could improve their effectiveness for intra‐articular treatment of OA, using a murine destabilization of the medial meniscus (DMM) model. Outcome parameters included voluntary gait activity, joint histology and RNA transcriptomic analyses of synovial tissues.

**Results:**

We found that activation of MSC via either innate immune pathway improved functional voluntary movement outcomes compared to treatment with non-activated MSC. Moreover, cartilage integrity, including cartilage preservation, was significantly improved in mice receiving activated MSC, with greater benefits observed in animals treated with STING pathway-activated MSC compared to animals treated with non‐activated MSC alone. Transcriptomic analysis of joint tissues revealed that treatment with activated MSC upregulated pathways associated with tissue remodeling, angiogenesis, and wound healing compared to tissues from animals treated with non-activated MSC.

**Discussion:**

These findings indicate therefore that innate immune activation of MSC prior to intra‐articular delivery for treatment of OA can significantly improve functional gait activity and chondroprotective effects compared to non‐activated MSC and suggest that this strategy could be evaluated clinically.

## Introduction

Osteoarthritis (OA) is a progressive, degenerative, condition that affects over 550 million people worldwide–a 113% increase since 1990. The prevalence has doubled in the US over the last 10 years with an economic impact of $136 billion annually (Yelin et al., 2016; Cui et al., 2020). Despite this high prevalence, there remains a lack of effective treatment options that improve quality of life without risk of adverse effects, with current therapies including non-steroidal anti-inflammatories, intra-articular injections, or arthroscopic debridement and cartilage resurfacing techniques. Cellular therapies to treat OA have emerged as an option in both human and veterinary orthopedics with mixed results reported in terms of efficacy (Mezey and Nemeth, 2015; Cortes-Araya et al., 2018; Ghannam et al., 2018; Kwon et al., 2022; Chahla et al., 2016; Harrell et al., 2019; Iijima et al., 2018; Li et al., 2018; Piuzzi et al., 2018; Şahin et al., 2018; Shah et al., 2018; Southworth et al., 2019; Xing et al., 2018). A recent meta-analysis of human randomized MSC trials concluded that overall MSC treatment reduced joint inflammation, improved pain scores, and preserved cartilage integrity, suggesting that MSC cellular therapy is beneficial. Nonetheless, challenges remain, including the use of MSC with greater potency for their intended task, greater uniformity of MSC sources and propagation, as well as improved credentialing of MSC functional properties that correlate with better outcomes (Copp et al., 2023). Heterogeneity within stromal cell populations has been proposed to be partially responsible for the observed variability in therapeutic responses, particularly in the context of variably inflamed recipient environments such as that seen in OA (Cassano et al., 2018). Pre-activation, or “inflammatory licensing,” of mesenchymal stromal cells (MSCs) through priming with their respective ligands has been proposed as a means to generate a homogenous population of immunomodulatory MSCs - thereby potentially improving the consistency of MSC therapy and response to treatment (Szabó et al., 2015; Krampera, 2011; Polchert et al., 2008; Waterman et al., 2012). With the lifetime likelihood to develop symptomatic knee OA currently at 45% and increasing, the need to develop improved strategies towards disease-modification is critical (Murphy et al., 2008).

Expression of pattern recognition receptors (PRRs), specialized proteins that detect exogenous pathogens and endogenous ligands, represent a key link between the immune system and sensory nervous system in response to inflammation or injury, such as that described in OA (Donnelly et al., 2020). The PRR family includes Toll-like receptors (TLR) (e.g., nucleotide oligomerization domain-like receptors, C-type lectin receptors, RIG-I-like receptors, and retinoic acid-inducible gene I receptors) and cytosolic DNA sensors (e.g., STimulator of Interferon Genes or STING). The STING receptor, also known as cGAS-STING, as Cyclic GMP-AMP synthase (cGAS), is the primary enzyme to induce STING pathway signaling. In response to tissue injury, PRRs on immune cells are activated to initiate their respective downstream inflammatory response. Activation of both STING and TLR3 results in production of type I interferons (IFN-Is) (IFN-α, IFN-β, and IFN-κ) in immune cells and sensory neurons following tissue injury or infection (Donnelly et al., 2021; Sun et al., 2022; Wang et al., 2021; Ishikawa and Glen, 2008; Liu et al., 2016), which has been demonstrated to have the potential to be both inflammatory or antinociceptive depending on the disease process (Donnelly et al., 2020; Liu et al., 2016). In the context of specifically enhancing therapeutic efficacy of immunomodulatory MSC therapy, our previous studies have demonstrated that stimulation of MSC with TLR ligands enhanced their immunomodulatory properties to a greater extent than other agonists evaluated, including cytokine secretion of MCP-1 and IL-8 *in vitro* resulting in resolution of inflammation and reduced bioburden associated with musculoskeletal infection in animal models (Pezzanite et al., 2021; Johnson et al., 2017; Pezzanite et al., 2022). We have more recently expanded this work to evaluate STING pathway agonism in MSC based on the concept that TLR3 and STING pathway agonists may trigger similar immune response pathways, particularly those involving interferon responses. However, to the authors’ knowledge, comparison of these two PRRs to activate and enhance MSC therapy for treatment of OA has not been previously reported.

Therefore, we built upon our previous work with TLR3 activated MSC to evaluate and compare the functional effects of activation of MSCs via TLR3 pathway and the cGAS-STING pathway in the context of intra-articular treatment of OA. The overall objective of this work was to determine whether priming of MSC with agonists of the cytoplasmic (endosomal) TLR3 pathway or the cytoplasmic cGAS-STING pathway exerted similar effects to improve the efficacy of MSC intra-articular therapy, as evaluated in a rodent destabilization of the medial meniscus (DMM) model of OA. Our analysis revealed significant improvements in post-operative voluntary movement parameters and histological outcomes with STING pathway activated MSC and improved histological outcomes with TLR3 pathway activated MSC. RNA sequencing of joint tissues indicated that IFN related pathways were upregulated in the joints of mice treated with either TLR3-activated MSC or STING-pathway activated MSC, with substantially more interferon pathways being upregulated in the STING-pathway MSC treated animals.

Overall, this work provides key new insights into the transcriptomic, structural, and synovial responses to intra-articular treatment with innate immune pathway activated MSC, suggesting that activation of TLR3 pathways or STING pathways in MSC prior to injection can activate multiple joint protective pathways, some of which may involve interferon gene pathway upregulation. Thus, stimulation of IFN pathways in MSC may be a uniquely effective method of licensing MSC for orthopedic applications.

## Materials and methods

This study was approved by the Institutional Animal Care and Use Committee at Colorado State University (IACUC protocol #3134) and conducted according to the national guidelines under which the institution operates and the NIH Guidelines for the Care and Use of Laboratory Animals (8th edition).

### Animals

**FIGURE 1:**
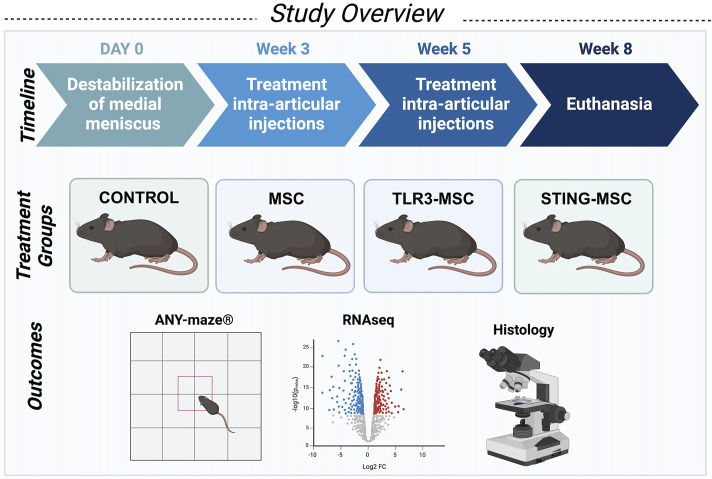
Schematic overview of study design. Studies were performed using 12-week-old male C57BL/6Nci mice (Charles River Laboratories, Wilmington, MA, United States). Mice (*n* = 10 per group for a total of forty mice) received unilateral destabilization of the medial meniscus surgery in the right knee and were randomly assigned to receive either control treatment (intra-articular needle insertion alone), non-activated MSC, PIC-activated MSC, or STING-activated MSC for two treatments at weeks three and five postoperatively. Activity monitoring was performed weekly on all mice in each group. At end term, five mice per group were assessed for histologic scoring and joint tissues from the remaining five mice were processed for transcriptomic analyses of joint tissues.

Studies were performed using 12-week-old male C57BL/6Nci mice (Charles River Laboratories, Wilmington, MA, United States). Mice (*n* = 10 per group) were randomly assigned to receive surgery followed by either (1) needle insertion alone, (2) non-activated MSCs, (3) pIC-activated MSCs (TLR3), or (4) 2′,3′ c-GAMP activated MSC (STING pathway), for a total of 40 mice. Activity monitoring was performed weekly on all mice in each group. At end term, five mice per group were assessed for histologic scoring of joint tissues, while the remaining five mice were processed for transcriptomic analyses of joint tissues. Mice were housed together in the Laboratory Animal Resources building in groups of five in solid bottom cages with corncob bedding and allowed *ad libitum* water and standard rodent chow. Mice were assessed by a veterinarian daily and body weights were monitored weekly. At the conclusion of the study, mice were euthanized via CO_2_ inhalation with confirmation by cervical dislocation. All animals were included in the final analyses. Study overview is provided in Figure 1.

### Stromal cell preparation

Age-matched male C57BL/6Nci mice served as adipose MSC (adMSC) donors. Murine adMSC was generated from abdominal and inguinal adipose tissue aseptically collected immediately following euthanasia via CO_2_ inhalation and cervical dislocation. The adipose tissue was isolated, pooled and cultured as previously described (Bunnell et al., 2008; Yoshimura et al., 2006). Cells generated were plastic-adherent and displayed typical adMSC morphology and expansion properties (Yoshimura et al., 2006). AdMSCs were expanded in culture in complete growth media (Dulbecco modified eagle medium (DMEM), 10% fetal bovine serum (FBS), penicillin (100 U/mL), streptomycin (100 μg/mL), and 1 mol/lL HEPES) until injection. MSCs are routinely evaluated for surface phenotype and found to be CD44^+^CD90^+^ and CD34^−^CD45^−^ in accordance with minimum criteria to define MSCs by the International Society for Cellular Therapy (Dominici et al., 2006). At weeks 3 and 5, MSC were trypsinized, counted, and a portion activated with the TLR3 agonist polyinosinic-polycytidylic acid (pIC) (InVivoGen, San Diego, CA) or 2′3′cGAMP (InVivoGen, San Diego, CA) to stimulate cGAS-STING pathways (stimulation dose at 10 μg/mL at a concentration of 1 × 10^6^ cells/mL in growth media for 2 h stimulation time). Cells were washed 3 times with sterile PBS and prepared at a concentration of 2.5 × 10^7^ cells per mL in PBS.

### Destabilization of the medial meniscus model of osteoarthritis

Osteoarthritis was induced in the right femorotibial joint (knee) of mice at time 0 (week 0) using established methods of destabilization of the medial meniscus (DMM) in the right hindlimb (Glasson et al., 2007). Mice were induced under general anesthesia using isoflurane (1%–5%, to effect) and the right femorotibial joint (knee) was clipped and aseptically prepared in routine fashion. Briefly, to induce OA, a medial parapatellar arthrotomy using a #11 scalpel blade was performed and the infrapatellar fat pad (IFP) temporarily repositioned laterally to allow access to the anterior medial meniscotibial ligament. This ligament was severed using the #11 scalpel blade. The IFP was repositioned, and the surgical incision closed in simple interrupted fashion using 6–0 monofilament absorbable suture. Mice were administered buprenorphine SR (slow release) 0.6–0.8 mg/kg subcutaneously under anesthesia at time of surgery.

### Intra-articular administration of adMSCs

Intra-articular injections of MSC were performed at weeks three and five following DMM operation. All mice received MSC from the same donor pool. For injections, mice were induced under general anesthesia using 3% isoflurane with oxygen, followed by 1%–1.5% isoflurane to maintain plane of anesthesia. The right (operated) femorotibial joint was aseptically prepared in routine fashion and injected with 2.5 × 10^5^ murine adipose-derived MSC or pIC- or STING-activated MSC suspended in 10 μL phosphate buffered saline (PBS), or controls of needle insertion alone. Injections were performed with #27 needle and Hamilton syringe. Although intra-articular MSC doses in humans have been reported to vary widely, the dose used here was determined based on scaling doses used in humans from human to murine body size and based on pilot studies indicating feasibility to achieve cell concentration within the volume injected (Copp et al., 2023). Dose of agonists were determined in previous pilot studies (data not shown) assessing differential gene expression and cytokine secretion following stimulation of MSC over a range of agonist doses, ranging from 0.01, 0.1, 1, to 10 μg/mL.

### Activity monitoring to assess osteoarthritis severity

Mice were monitored prior to injury and for 8 weeks following surgery using individual cage monitoring to determine general animal behavior and mobility. Cage monitoring was performed for 10 min weekly during the experimental time-course. Mice were placed in their primary enclosure/resident cage with their environmental enrichment hut for the duration of the assessment. Prior to taking the baseline measurement, mice were acclimated to the system over 1 week - after which two baseline measurements were collected immediately before the start of the study. Training and data collection occurred during the same time of day (8 a.m.–12 p.m.) and involved the same handlers throughout the course of the study to minimize circadian rhythm cycle variations. The video analysis software used (ANY-maze™, Wood Dale, IL, United States) automatically collected mobility parameters including total distance traveled, time mobile, mean speed, maximum speed, time in hut, and entries to the top of the hut. Parameters of interest were assessed both cross-sectionally amongst groups normalized to preoperative baseline and longitudinally over time.

### Transcriptomic sequencing of joint tissues

Mouse femorotibial (knee) tissues (*n* = 5 biological replicates per treatment group of 10 mice) were removed *en bloc* (including all joint tissues such as cartilage, synovium, joint capsule, and adjacent subchondral bone of the femur and tibia) immediately following euthanasia at 8 weeks after surgery, minced, and stored in RLT lysis buffer (Qiagen) at −80°C. RNA was extracted by first using TRIzol Reagent (Invitrogen, Waltham, MA) following manufacturers’ instructions, with a 1:3 RLT lysis buffer to TRIzol ratio. RNA precipitate pellet was then cleaned and concentrated using RNeasy MinElute Cleanup Kit (Qiagen Germantown, MD), according to manufacturer’s instructions and sent to Novogene Corporation Inc. (Sacramento, CA) for bulk RNA sequencing. RNA quality was determined by bioanalyzer (Agilent Technologies, Santa Clara, CA). RIN (RNA integrity number) was determined to be >7.5 for all samples. For bulk RNA sequencing, mRNA was enriched using oligo (dT) beads, followed by cDNA library generation using TruSeq RNA Library Prep Kit (Illumina, San Diego, CA). Sequencing was performed on Illumina Novaseq 6000 machine using 150 bp paired end reads.

### Joint histology

At 8 weeks after surgery, femorotibial (knee) joints (*n* = 5 biological replicates per treatment group of 10 mice) were fixed in 10% neutral buffered formalin for 24 h, then decalcified in ethylenediamine tetra-acetic acid (EDTA) and paraffin embedded. Coronal sections (5 µm) were taken from the center of the medial tibial plateau and stained with toluidine blue. Histological grading of joint tissues was performed using an established criteria for OA by two blinded, independent individuals trained in using this scoring method via consensus. In brief, joint tissues were semi-quantitatively graded for osteoarthritic damage including cartilage fibrillation, cartilage loss including clefts/erosions and calcification, synovitis, and proteoglycan content for the whole joint and medial and lateral joint compartments (Haubruck et al., 2023).

### Data analysis

The experimental sample size was calculated by *a-priori* power analysis using GPower Version 3.1.1, using pilot gait data (stride length) obtained using this injury model in mice as the primary outcome measure for calculating group sizes. Cross-sectional activity monitoring data was normalized to baseline values and compared using repeated-measures analysis of variance (ANOVA) with Tukey’s correction. Longitudinal data were compared using repeated-measures ANOVA and Dunnett’s correction, comparing each timepoint to baseline values. Histological scoring at end-term was evaluated using a one-way non-parametric ANOVA (Kruskal–Wallis test) with Dunn’s multiple comparisons test. Statistical analysis was performed using GraphPad Prism v9.3.1 (GraphPad Software Inc., La Jolla, CA, United States). The significance for enclosure monitoring and histological outcomes was assessed at *p* < 0.1 as described (Jennions and Moller, 2003).

Sequence data were analyzed on Partek Flow software, version 10.0 (Partek Inc. Chesterfield, MO). First, Raw data were filtered by removing reads containing adapters and reads containing N > 10% and for Phred scores >30. Filtered reads were then aligned with STAR 2.7.3a using GRCm39 Genome Reference. Aligned reads were annotated and counted using HT-seq with Ensembl 108. Differentially expressed genes were identified using DEseq2 (Love et al., 2024). Biological interpretations included gene ontology and gene set enrichment analysis (GSEA), which were performed using GSEA (https://www.gsea-msigdb.org/gsea/index.jsp).

## Results

### Comparison of functional impact of treatment via activity monitoring

**FIGURE 2:**
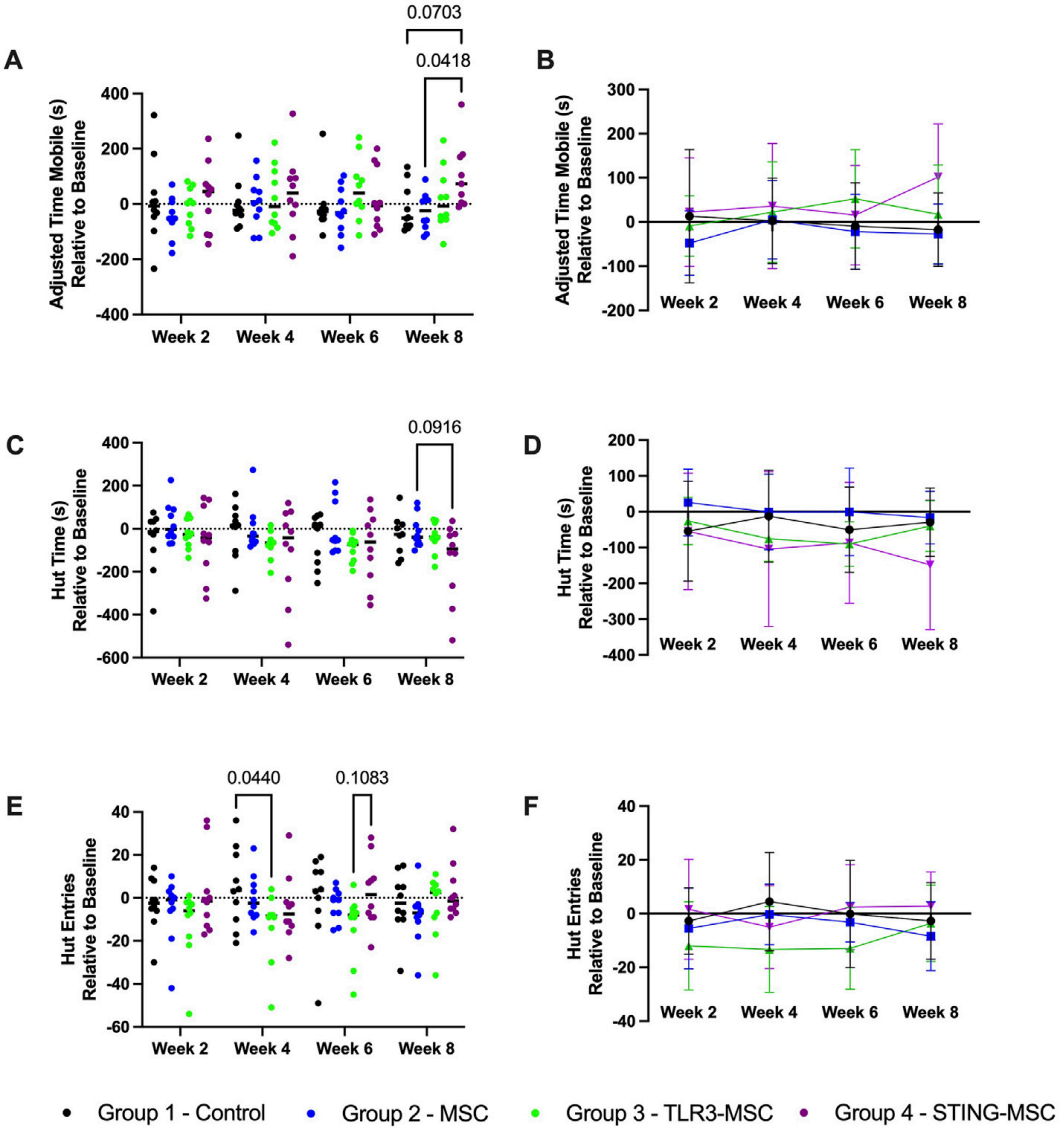
ANY-maze^TM^ cage monitoring parameters. Individual cage activity monitoring was performed following DMM surgery on the right hind limb of mice and two intra-articular treatments administered depending on group. Parameters of interest are shown over time and between groups for adjusted time mobile (s) **(A,B)** relative to baseline, hut time (s) relative to baseline **(C,D)** and hut entries relative to baseline **(E,F)**. Significant differences noted, with *p*-value noted as <0.1 and significant differences labeled. TLR, Toll-Like receptor; STING, Stimulator of Interferon Genes; DMM, destabilization of medial meniscus; m, meters; s, seconds.

We assessed functional improvement with treatment over time using the ANY-maze overhead cage monitoring software. When cross-sectional data was normalized to pre-DMM surgery baseline values, mice treated with STING pathway activated MSC exhibited greater adjusted time mobile compared to MSC treated mice (*p* = 0.04) or control (*p* = 0.07) at week 8 (Figures 2A,B). In addition, mice treated with STING-MSC spent less time in their security huts relative to baseline compared to MSC treated mice (*p* = 0.09) at week 8 (Figures 2C,D), and fewer hut entries relative to baseline compared to control at week 4 (*p* = 0.04) or TLR3-pathway activated MSC treated mice at week 6 (*p* = 0.1) (Figures 2E,F). There were no significant differences between TLR3 MSC treated mice compared to needle or MSC alone. There were no significant findings in longitudinal data for within group (e.g., time) differences (Figure 2 significant findings, Supplementary Table S1, additional non-significant findings, Supplementary Table S2, tracking pathways).

### Joint immune transcriptome responses to intra-articular therapy

To further understand how intra-articular injection affected the overall immune transcriptome of the joint, mice were treated by intra-articular injection of activated or non-activated MSC, and 24 h later RNA was extracted from harvested joint tissues (pooled tissues included cartilage, synovium, joint capsule and adjacent subchondral bone of femur and tibia) and analyzed via bulk RNA sequencing. Comparisons between the transcriptomes of stifle (knee) joints that were treated with STING- pathway activated -MSC compared to MSC, TLR3-pathway activated-MSC versus MSC, and STING pathway activated-MSC versus TLR3 pathway activated-MSC were performed and described below. Additional comparisons between each treatment group to control needle insertion alone are provided in Supplementary Figure S1. Full gene set enrichment analysis (GSEA) is provided in Supplementary Figure S2.

**FIGURE 3:**
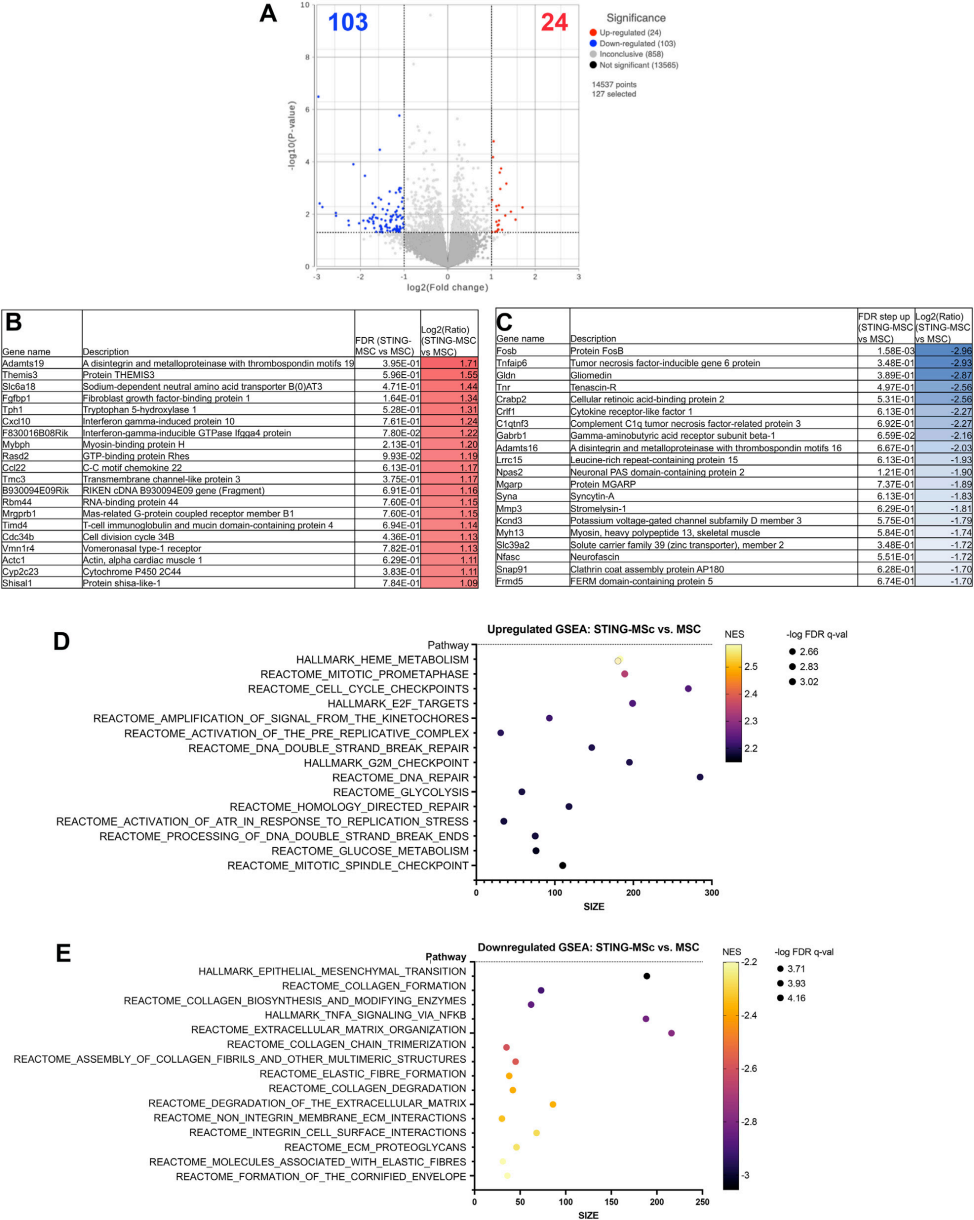
RNA sequencing analysis comparing transcriptomes of joint tissues from mice treated with STING-MSC versus MSC. Volcano plot of transcriptome from *n* = 5 biological replicates of STING-MSC versus MSC treated mouse knee joints **(A)**. X-axis shows fold change and y-axis shows FDR adjusted *p*-value, with significantly upregulated genes shown as red dots and significantly downregulated genes shows as blue dots. Significance defined as FDR ≤0.05 fold change ≥2 or ≤−2. Differential gene expression (list of genes, description, unadjusted *p*-value and fold-change of top 20) for upregulated **(B)** and downregulated **(C)** genes in differential analysis results from STING-MSC versus MSC treated joints. Pathway analyses of differential gene expression were performed using normalized counts from *n* = 5 biological replicates of mice treated with either STING-MSC or MSC alone, indicating the top upregulated **(D)** and downregulated **(E)** pathways. FC, Fold Change; FDR, false discovery rate; MSC, mesenchymal stromal cells; TLR, Toll-Like receptor; STING, Stimulator of Interferon Genes.

Comparison of knee joint tissues from STING-pathway activated-MSC to MSC treated animals demonstrated significant upregulation of 24 genes and downregulation of 103 genes (significance defined as fold change ≥2 or ≤ −2 or *P* value ≤ 0.05) (Figure 3A). Differential gene expression (list of genes, description, unadjusted *p*-value and fold-change of top 20) for upregulated (Figure 3B) and downregulated (Figure 3C) genes in differential analysis results are reported from STING pathway activated-MSC versus MSC treated joints. Pathway analyses show the top 15 upregulated (Figure 3D) and downregulated (Figure 3E) pathways.

**FIGURE 4:**
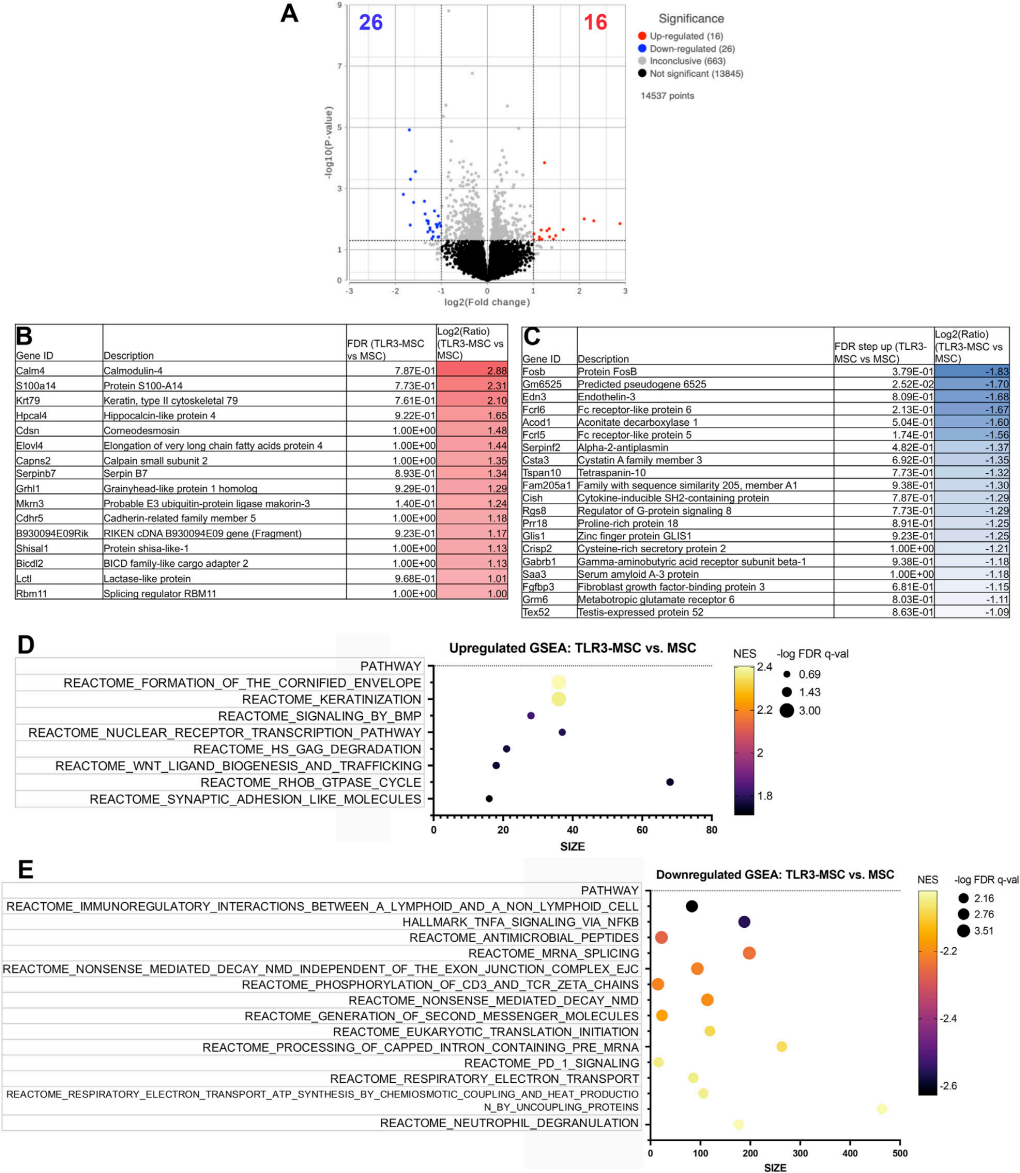
RNA sequencing analysis comparing transcriptomes of joint tissues from mice treated with TLR3-MSC versus MSC. Volcano plot of transcriptome from *n* = 5 biological replicates of TLR3-MSC versus MSC treated mouse knee joints **(A)**. X-axis shows fold change and y-axis shows FDR adjusted *p*-value, with significantly upregulated genes shown as red dots and significantly downregulated genes shows as blue dots. Significance defined as FDR ≤0.05 fold change ≥2 or ≤−2. Differential gene expression (list of genes, description, unadjusted *p*-value and fold-change of top 20) for upregulated **(B)** and downregulated **(C)** genes in differential analysis results from TLR3-MSC versus MSC treated joints. Pathway analyses of differential gene expression were performed using normalized counts from *n* = 5 biological replicates of mice treated with either TLR3-MSC or MSC alone, indicating the top upregulated **(D)** and downregulated **(E)** pathways. FC, Fold Change; FDR, false discovery rate; MSC, mesenchymal stromal cells; TLR, Toll-Like receptor; STING, Stimulator of Interferon Genes.

Similar findings were also noted in joint tissues of mice treated with TLR3 pathway activated MSC. For example, Comparison of knee joint tissues from TLR3 pathway activated-MSC to MSC treated animals demonstrated significant upregulation of 16 genes and downregulation of 26 genes (significance defined as fold change ≥2 or ≤ −2 or *P* value ≤ 0.05) (Figure 4A). Differential gene expression (list of genes, description, unadjusted *p*-value and fold-change of top 20) for upregulated (Figure 4B) and downregulated (Figure 4C) genes in differential analysis results from TLR3-MSC versus MSC treated joints are reported. Pathway analyses highlighted the top 15 upregulated (Figure 4D) and top 15 downregulated (Figure 4E) pathways.

**FIGURE 5:**
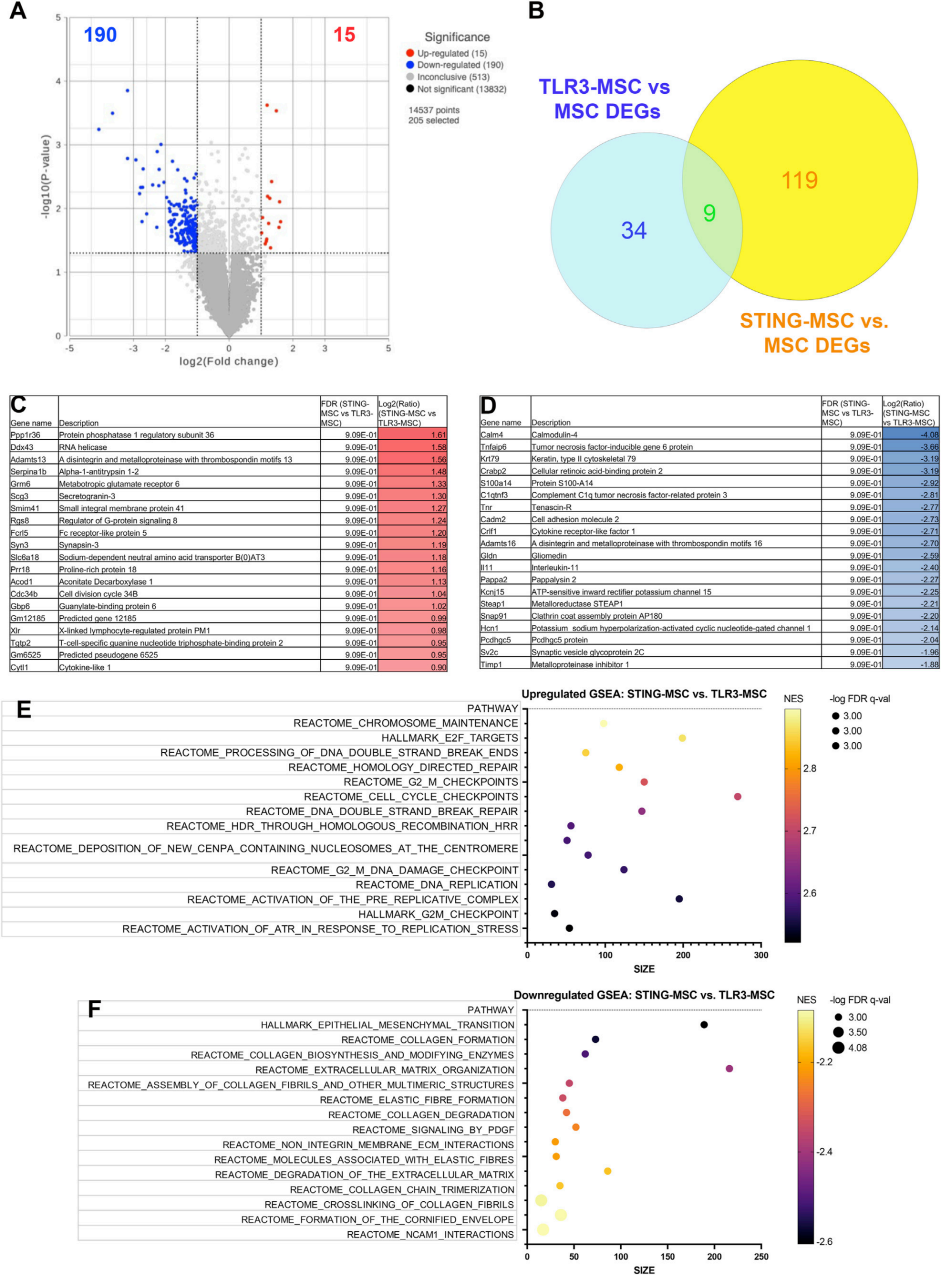
RNA sequencing analysis comparing transcriptomes of joint tissues from mice treated with STING-MSC versus TLR3-MSC. Volcano plot of transcriptome from *n* = 5 biological replicates of TLR3-MSC versus MSC treated mouse knee joints **(A)**. X-axis shows fold change and y-axis shows FDR adjusted *p*-value, with significantly upregulated genes shown as red dots and significantly downregulated genes shows as blue dots. Significance defined as FDR ≤0.05 fold change ≥2 or ≤−2. Venn diagram of differentially expressed genes for each comparison group and to resting MSC **(B)**. Differential gene expression (list of genes, description, unadjusted *p*-value and fold-change of top 20) for upregulated **(C)** and downregulated **(D)** genes in differential analysis results from TLR3-MSC versus MSC treated joints. Pathway analyses of differential gene expression were performed using normalized counts from *n* = 5 biological replicates of mice treated with either TLR3-MSC or MSC alone, indicating the top upregulated **(E)** and downregulated **(F)** pathways. FC, Fold Change; FDR, false discovery rate; MSC, mesenchymal stromal cells; TLR, Toll-Like receptor; STING, Stimulator of Interferon Genes.

Direct comparison of knee joint tissues from STING pathway activated-MSC versus TLR3 pathway activated-MSC treated animals demonstrated significant upregulation of 15 genes and downregulation of 190 genes (significance defined as fold change ≥2 or ≤ −2 or *P* value ≤ 0.05) (Figure 5A). A Venn diagram of differentially expressed genes for each comparison group is illustrated in Figure 5B. Differential gene expression (list of genes, description, unadjusted *p*-value and fold-change of top 20) for upregulated (Figure 5C) and downregulated (Figure 5D) genes in differential analysis results from STING-MSC versus TLR3-MSC treated joints indicating the top 15 upregulated (Figure 5E) and downregulated (Figure 5F) pathways.

### Impact of treatment on histologic joint scoring

**FIGURE 6:**
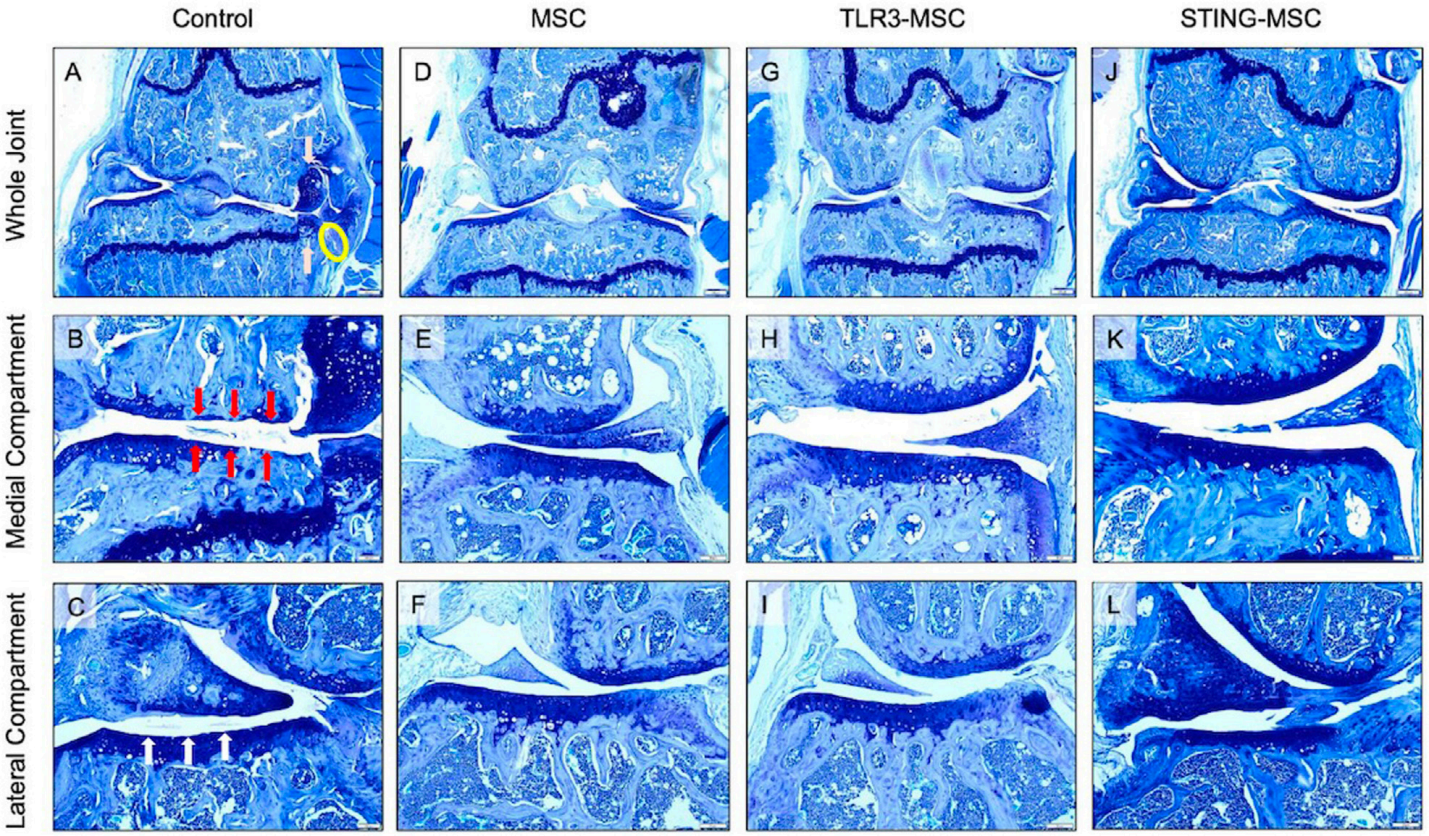
Histologic representative images of mouse knees at endterm. Toluidine blue photomicrographs from (left to right) control (needle insertion alone), MSC, TLR3-MSC, and STING-MSC treated (right) limbs. Low magnification (×4) images of whole knee joints from (left to right) control **(A)**, MSC **(D)**, TLR3-MSC **(G)**, and STING-MSC **(J)** treated joints. Higher magnification (×10) images presented for evaluation of the medial compartment of (left to right) control **(B)**, MSC **(E)**, TLR3-MSC **(H)**, and STING-MSC **(K)** treated joints. Additional higher magnification image for evaluation of the lateral compartment for control **(C)**, MSC **(F)**, TLR3-MSC **(I)** and STING-MSC **(L)** treated joints. **(A,D,G,J)** 4x, scale bar = 200 μm; **(B,C,E,F,H,I,K,L)** 10x, scale bar = 20 μm. Pink arrows represent mostly cartilagenous osteophytes. The yellow circle indicates synovitis. Red arrows highlight areas of cartilage loss to below the calcified layer. White arrows outline a roughened cartilage surface, as well as fibrillation. MSC, mesenchymal stromal cells; TLR, Toll-Like receptor; STING, Stimulator of Interferon Genes.

We also evaluated the impact of intra-articular treatment on joint pathology according to an updated quantitative joint-wide histopathological scoring system developed for murine post-traumatic osteoarthritis models (Haubruck et al., 2023). Medial and lateral scores were assigned for synovitis scores and for the femur and tibia individually, which were combined to obtain the overall medial and lateral scores that were added to achieve the total joint score. In animals that underwent the DMM procedure but with no treatment, there was synovitis, fibrillation, clefts in the medial and/or lateral compartments, and, in some instances, osteophytes on the medial aspect of the joint (representative images, Figure 6).

**FIGURE 7:**
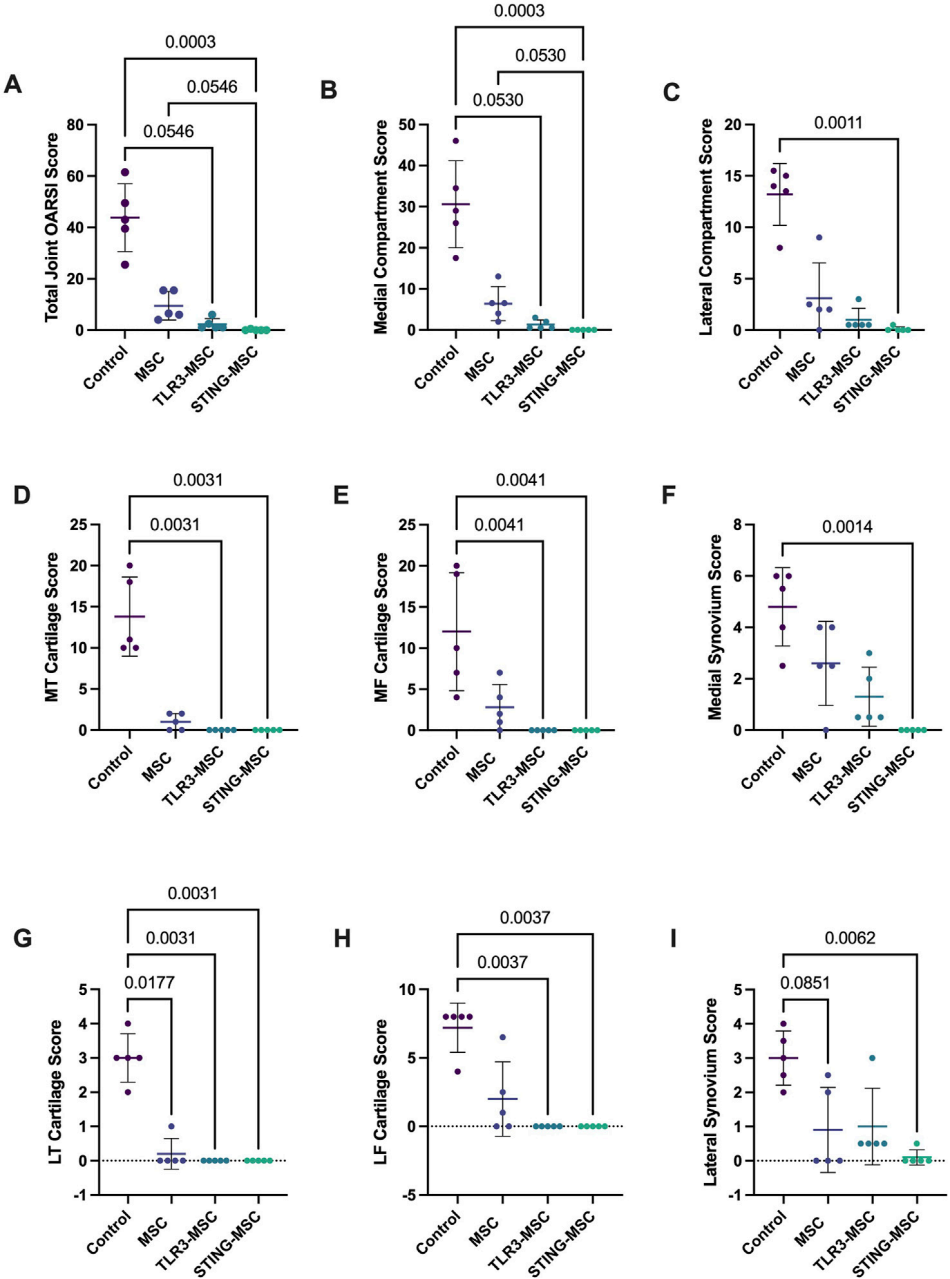
OARSI histopathology scores. Scoring of histology slides was performed following DMM surgery and treatment with control (needle insertion alone), MSC, TLR3-MSC, or STING-MSC. Parameters listed include whole joint OARSI total score **(A)**, medial compartment total score **(B)**, lateral compartment total score **(C)**, medial tibial cartilage score **(D)**, medial femoral cartilage **(E)**, medial synovium score **(F)**, lateral tibial cartilage score **(G)**, lateral femoral cartilage score **(H)**, lateral synovium score **(I)**. Significant differences noted with *p* values < 0.05. MSC, mesenchymal stromal cells; TLR, Toll-Like receptor; STING, Stimulator of Interferon Genes.

Total and individual compartment joint scores were significantly improved by treatment with non-activated MSC. Specifically, we observed that compared to treatment with non-activated MSC, treatment with either TLR3 or STING pathway activated MSC exerted a significantly greater improvement in overall joint structure. For example, Overall joint scores (*p* = 0.0003, *p* = 0.05) and medial compartment scores (*p* = 0.0003, *p* = 0.05) were improved in STING-MSC treated joints compared to control and MSC-treated, respectively (Figures 7A,B). Lateral compartment scores were also improved in STING-MSC treated vs control joints (*p* = 0.001) (Figure 7C). Overall joint and medial compartment scores (*p* = 0.05, *p* = 0.05) were improved in TLR3-MSC treated joints compared to control joints. Additional scored and individual OARSI parameters for tibia, femur, and synovium in the medial (Figures 7D–F) and lateral compartments (Figures 7G–I) are presented. These findings indicate that activation of MSC with either of two different innate immune pathway agonists exerted an important positive impact on the effectiveness of the MSC for improvement of overall joint structure and function following PTOA, compared to treatment with non-activated MSC.

## Discussion

Intra-articular mesenchymal stromal cell products have demonstrated therapeutic potential in preclinical models of OA previously with varied results reported for efficacy. The findings reported here add to the body of literature regarding the use of regenerative therapies to treat OA, highlighting the potential for STING agonism of MSCs to enhance both functional outcomes and structural joint integrity while promoting transcriptional pathways favoring tissue repair. This was demonstrated via significant improvement in voluntary movement parameters (adjusted time mobile, maximum speed, time spent in hut) (Figure 2) and differential gene expression in joint tissues favoring a tissue healing environment with upregulated genes associated with remodeling, cell proliferation and angiogenesis (Figures 3–5). Importantly, all MSC treated groups also showed improved cartilage integrity and synovial scores compared to control animals (Figures 6, 7).

Pattern recognition receptors (PRRs) play a key role in immune and sensory nervous system regulation of pain and inflammation in injury (Rodriguez-Palma et al., 2024). Following tissue damage, PRRs on immune cells are activated to initiate an inflammatory response and sensory neurons concurrently sense these signals PRR expression themselves. In this environment, stimulator of IFN genes (STING) has been recognized as a regulator of pain signaling, where activation of STING receptors has been demonstrated to result in production of type I IFNs in immune cells and sensory neurons and subsequently to elicit antinociceptive effects in neuropathic mice (Donnelly et al., 2021; Sun et al., 2022; Wang et al., 2021; Ishikawa and Glen, 2008). Conversely, deletion of the STING gene has been shown to result in mechanical allodynia (Donnelly et al., 2021). While seemingly counterintuitive to induce production of pro-inflammatory mediators associated with further pain propagation, recent evidence indicates that inflammation can contribute to resolution of pain through further induction of counter-regulatory anti-inflammatory mediators (Sugimoto et al., 2016). Studies exploring the STING pathway elicited type I IFN (INF-α, IFN-β) signaling to alleviate pain have yielded conflicting results, indicating STING agonism and type I IFN production can be both pro- and anti-inflammatory depending on the context in which they are evaluated. For example, STING agonism has produced antinociceptive effects in the central nervous system while STING agonists or recombinant IFN-α injected peripherally have also elicited nociceptive behaviors suggesting pain induction (Liu et al., 2016; Barragan-Iglesias et al., 2020; Fitzgibbon et al., 2019; Franco-Enzastiga et al., 2024). In a rodent model of inflammatory pain, inflammation-induced activation of STING in dorsal root ganglia nociceptors reduced pain-like behaviors (Defaye et al., 2024). Using a gain-of-function STING mutation model, INF-α regulation of voltage-gated potassium channels was identified as the mechanism behind this observed reduction in pain (Defaye et al., 2024). In this study, as the STING-activated MSC themselves likely only persist transiently in the joint, their demonstrated mechanism of action is likely to result from paracrine effects lasting long after the innate immune activated MSC have been cleared. Potential paracrine effects revealed by the transcriptomic studies (Figures 3–5) of joint tissues from animals treated with either TLR3 or STING pathway activated MSC include induction of anabolic processes within joint tissues, suppression of ongoing joint inflammation, and stimulation of joint reparative processes. In addition, cytokines (especially IFNs) secreted by the innate immune activated MSC may have elicited activation of immune counter-regulatory immune responses that further suppressed joint inflammation.

When comparing differentially expressed genes and transcriptomic pathway analyses between treatment groups in this study, treatment of joints with STING pathway activated MSC resulted in upregulation of gene pathways associated with cell cycle activation, proliferation, DNA damage response and repair, and downregulation of genes associated with extracellular matrix, most likely driven by osteoclast, synovial fibroblasts or chondrocytes. Several gene signatures emerged as being potentially particularly relevant to how these two immune pathways (TLR3 and STING pathways) might be functioning to improve joint function. Particularly in the downregulated pathways, significant overlapping gene sets were found between the STING and TLR groups. For example, downregulation of inflammatory and cytokine signaling (biocarta TNFR2, Hallmark IL2 STAT5, Hallmark TNFA, Hallmark inflammatory response) (Supplementary Figure S2); as well as downregulation of cellular stress and senescence (Hallmark hypoxia, p53, UV response and Reactome oncogene induced senescence). Suppression of senescence in fact has been a much-explored area in the reversal of joint damage and treatment of arthritis (Han et al., 2024; Jinjin et al., 2022). HIF1a (Hypoxia-inducible factor-1alpha) and other hypoxia associated genes are also linked to OA related degeneration (Zhang et al., 2025; Zhang and Kong, 2023).

As discussed, IFN-related genes have been previously implicated in the resolution of inflammatory pain (Love et al., 2024), although the role of type I IFNs in this regard remains controversial with reports supporting both pro- and anti-nociceptive actions of IFN-α and IFN-β (Tan et al., 2021). Other potential functions of upregulated IFN pathways in slowing joint degeneration include stimulation of counter-regulatory immune pathways (e.g., IDO pathway, PD-L1 and other checkpoint molecule expression, expansion of Tregs, recruitment of immune suppressive monocytes), all of which may contribute to reducing joint inflammation and preserving cartilage integrity, and suppression of deleterious vascular responses by suppressing abnormal angiogenesis (Rodriguez-Palma et al., 2024). Importantly, a recent publication also highlighted the role of IFN-g in stimulating cartilage regeneration (Kim et al., 2024). Other IFN-regulated pathways may also be involved in improved joint function following injection of activated MSC. For example, alterations in tryptophan metabolism have been previously associated with occurrence and development of OA (Yang et al., 2024). In joints of STING pathway activated-MSC treated mice, one of the most upregulated genes was encoding tryptophan 5-hydroxylase, an enzyme essential to synthesis of the neurotransmitter serotonin. Tryptophan metabolite disturbances have been associated with erosive hand osteoarthritis in people (Binvignat et al., 2023) and conversely, supplementation of tryptophan metabolites including 5-hydroxytryptophan have been shown to suppress inflammation and arthritis through suppression of pro-inflammatory mediator production in a rodent inflammatory collagen-induced arthritis model (Yang et al., 2015). In this study, we observed enhanced mobility in animals treated with immune activated MSC suggesting alleviation of pain, limited structural disease progression in joints evidenced by improved histologic scores (Figures 6, 7). Further investigation into the mechanisms by which induction of inflammatory pathways with STING agonism reduces pain and limits disease progression in the context of OA may lead to new insights to improve our approaches to treat chronic persistent musculoskeletal pain in animals and humans with a variety of different types of OA.

Loss of mobility and articular associated pain are primary reasons for individuals with OA to seek treatment. Therefore, cage monitoring, or “open field testing” of functional activity which we performed here can offer insights as to voluntary behavior and mobility differences relative to baseline as an indication of pain following injury. In this study, by 8 weeks post-DMM surgery, mice injected with two doses of STING-MSC demonstrated increased activity, evidenced by greater adjusted time mobile and less time in their security huts, implying potential differences in clinical or pain responses between groups (Figure 2). Additionally, STING-MSC treated animals exhibited the greatest number of entries to the top of their hut compared to other treatment groups, which reached significance compared to TLR-MSC treated mice at week 6. In this hindlimb injury model, this may indicate an increased willingness to climb, use and propel off the injured limb to enter the top of the hut, potentially indicating earlier return to full hind limb function. It is acknowledged that while cage monitoring or open-field testing in rodent models implies pain following injury, these methods to assess voluntary behavior do not necessarily correlate to pain or mobility experienced by humans. However, the findings reported here do support previous studies in humans where intra-articular cellular based therapies have been reported to decrease pain and improve functionality in knee OA, resulting in increased physical wellness overall (El-Kadiry et al., 2022; Anz et al., 2020).

Histopathology remains the gold standard in animal models to assess structural disease in OA pathology characterized by progressive articular cartilage degradation and osteophyte development (Zaki et al., 2022; Newman et al., 2022; Aigner et al., 2010). In small animal (i.e., rodent) species, evaluation of the entire joint in a single plane allows simultaneous examination of the entire joint, including pathological changes in cartilage degradation, synovial inflammation and fibrosis, meniscal tearing and bone remodeling including marginal osteophytes or enthesiophytes and subchondral sclerosis. Multiple scoring systems have been reported for histopathological evaluation of the mouse knee OA (Glasson et al., 2010; Chambers et al., 1997; Little et al., 2009; McNulty et al., 2011; Sampson et al., 2011; Pinamont et al., 2020; Armstrong et al., 2021), with the destabilization of the medial meniscus (DMM) model presented here representing a reproducible animal model that allows longitudinal evaluation of disease. Haubruck et al. further developed a semi-quantitative mouse OA histopathology scoring system to grade cartilage as well as synovium, meniscus, cruciate ligaments, subchondral bone and marginal osteophytes/enthesiophytes, demonstrating evaluation of a single slide allowed reproducible grading of histopathological changes in joint tissues in this model and reduced scoring time without compromising sensitivity and specificity of results (Haubruck et al., 2023). Using this scoring system, here we notably demonstrated significant improvement in total joint and medial compartment scores with STING pathway activated-MSC treatment compared to control (needle insertion alone) and non-activated MSC, as well as improvement in the lateral compartment between STING pathway activated-MSC to control. When medial and lateral compartments were examined separately, we further demonstrated significant improvement between STING pathway activated-MSC versus control for both medial and lateral tibial and femoral cartilage and synovium scores. TLR3 pathway activated-MSC therapy also resulted in improvement, albeit to a lesser extent compared to STING pathway activated-MSC in overall joint and medial compartment scores compared to needle insertion alone. As animal models remain the foundation for development of therapeutic interventions for human OA with histopathology considered the gold standard for assessment, these findings underscore the potential for immune activated cellular therapy, particularly STING pathway-activated MSC, to mitigate structural disease progression.

Limitations to this study include the relatively short study duration (8 weeks), although this has been reported and considered typical for this model of OA previously. The mouse DMM model was selected as the first *in vivo* model to initially screen the immune-licensed MSC treatment described due to the low cost of working with rodent models, high reproducibility of the model, fewer/more localized cartilage defects compared to other rodent models (e.g., anterior cruciate ligament transection) making it easier to assess treatment effect, and slower disease progression that more closely mimics human OA, allowing for relatively consistent evaluation of pain-associated behaviors (Glasson et al., 2007; Bapat et al., 2018; Hu et al., 2022). Although significant differences in gait parameters and histopathologic findings were seen between treatment groups by 8 weeks postoperatively, additional information may have been gained by allowing OA to progress for a more prolonged period. Additionally, mice in this study were operated and evaluated following skeletal maturity at 12 weeks of age which would simulate post-traumatic OA in an early adult population (i.e., mid-twenties); however, it is recognized that knee OA in humans frequently occurs in middle age to elderly patients and further evaluation of this treatment strategy at later age time points would be valuable. The lack of sham-operated controls is acknowledged as a study limitation; however, this work was designed to specifically assess acute changes following cell injection and impact of activation procedures rather than further delineating the DMM model.

Furthermore, this study evaluated outcomes following two MSC injections at 3- and 5-weeks post-injury, the timing of which was based upon initial pilot studies (not shown). The MSC used in this study underwent minor phenotypic characterization and it is acknowledged that more detailed MSC phenotyping may be required to sufficiently replicate these data due to heterogeneity in MSC populations and potential variation in isolation protocols between laboratories. Further assessment of injection number, timing of injection(s) during the temporal course of disease progression and dose with an expanded examination of control groups may demonstrate greater improvement and/or provide further information as to the effect of the immune-licensed cellular products. Addition of treatment groups injecting the MSC secretome or extracellular vesicles (EVs) derived from of these immune-activated MSCs were not within the scope of this study; however, evaluation of the impact of immune licensing on immunosuppressive function of EVs or secreted products generated under different culture methods (e.g., 3D vs. 2D monolayer culture) represent future directions of this work. Finally, the use of fetal bovine serum (FBS) in growth media during isolation, culture, and expansion of MSC before immune licensing is acknowledged as a limitation; while the use of FBS in preclinical models is accepted, evaluation of alternate serum-free sources is warranted prior to future translational applications. The findings herein warrant further investigation in additional studies designed to elucidate more specific mechanisms underlying the effect and expanding to large animal models of OA.

In summary, these findings provide key insights into the functional, transcriptomic, and histologic synovial response to intra-articular immune activated (pIC or 2′,3′-cGAMP) activated MSC therapy. Notably, all three MSC therapeutic approaches improved histologic outcomes in this model, while STING-pathway activated-MSC treatment and TLR3-pathway activated MSC resulted in further improvement in key functional parameters and induced transcriptomic changes promoting tissue repair. These findings provide further mechanistic insight as to possible mode of action through which STING and TLR3 pathway activated-MSC may be exerting a therapeutic effect in early post-traumatic OA, relating differential gene expression on the cellular level to improvement seen functionally and structurally on histology. Thus, there is precedent for multiple different IFN pathways being critically involved in reducing joint inflammation and improving mobility and cartilage integrity. These findings suggest that pre-activation of MSC with stimuli that induce strong IFN responses could be an successful strategy to improve the overall effectiveness of cellular therapy for OA.

## Data Availability

The datasets presented in this study can be found in online repositories. The names of the repository and accession number can be found below: GEO public genomic data repository, with accession number GSE276262.

## Supplementary material

SUPPLEMENTARY TABLE S1 Anymaze cage monitoring data for non-significant parameters of interest.

SUPPLEMENTARY TABLE S2 Anymaze track pathways demonstrating variability in mobility among treatment groups.

SUPPLEMENTARY FIGURE S1 RNA sequencing analysis comparing transcriptomes of joints treated with MSC, TLR3-MSC or STING-MSC to control (needle insertion alone).

SUPPLEMENTARY FIGURE S2 Complete summary of gene set enrichment analysis (GSEA) of pathways and scoring.
