# Supplementary figures and images for: Innate immune pathway activated mesenchymal stromal cells improve function and histologic outcomes in a rodent osteoarthritis model
Source: Front Bioeng Biotechnol. 2025 May 22;13:1525969. doi: 10.3389/fbioe.2025.1525969 (PMC12138259; doi:10.3389/fbioe.2025.1525969)

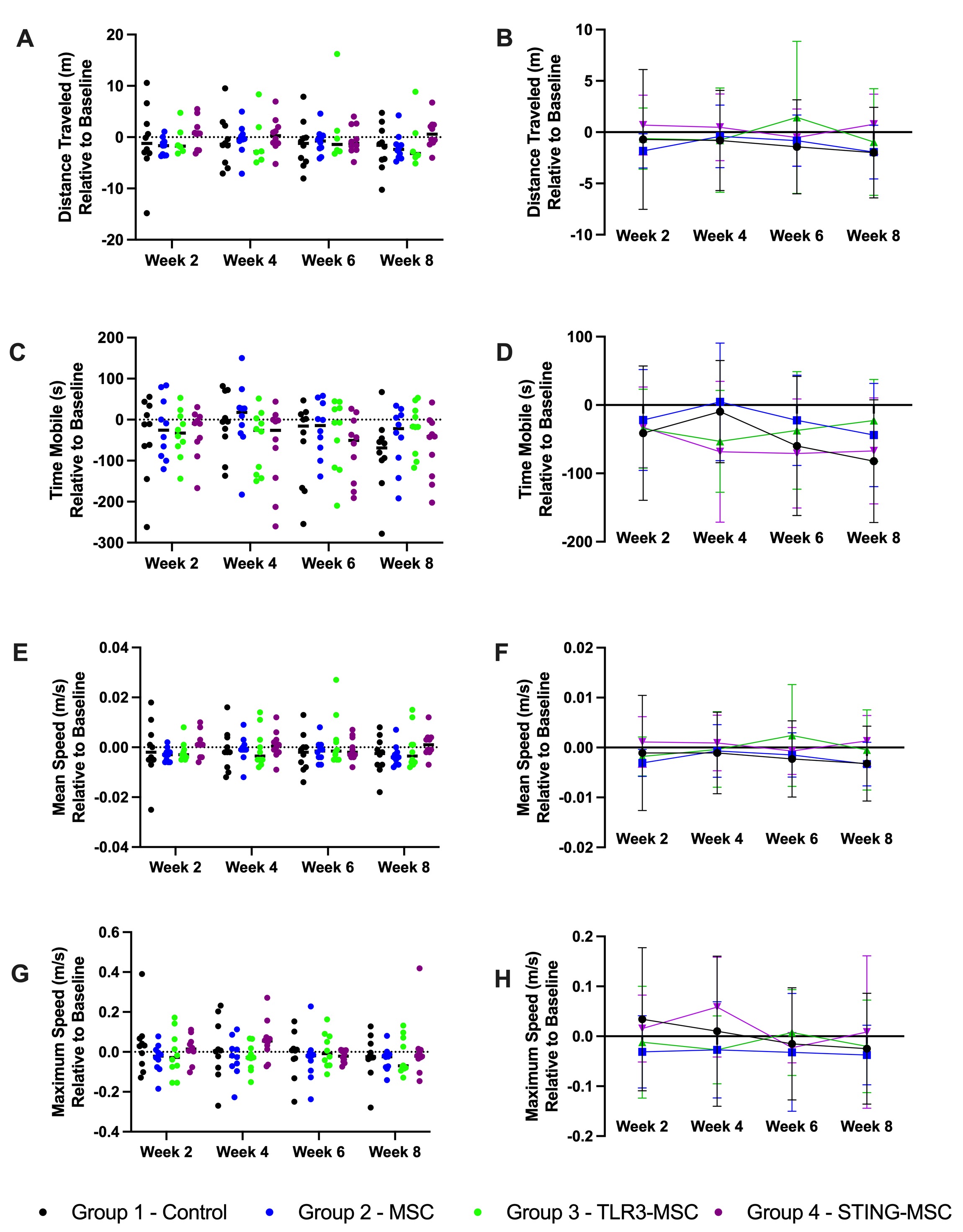

Supplement: Supplementary file 2 [file Image1.jpeg]

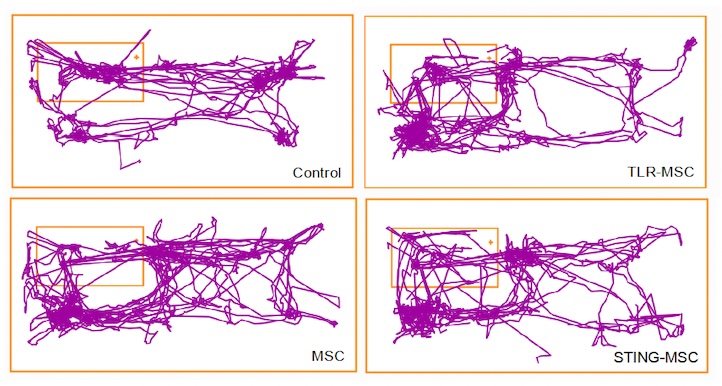

Supplement: Supplementary file 3 [file Image2.jpeg]
